# A rare case report of panuveitis with retinochoroidal involvement, retinitis, and retinal vasculitis due to extensive tinea corporis

**DOI:** 10.3389/fopht.2023.1174414

**Published:** 2023-06-09

**Authors:** Vinaya Kumar Konana, Kalpana Babu

**Affiliations:** Department of Uveitis and Ocular Inflammation, Vittala International Institute of Ophthalmology and Prabha Eye clinic and Research Centre, Bangalore, Karnataka, India

**Keywords:** panuveitis, retinitis, tinea corporis, itraconazole, skin, retinal vasculitis

## Abstract

A 40-year-old Asian Indian woman, diagnosed as having idiopathic panuveitis (elsewhere) 3 years earlier and being treated with oral steroids (20 mg/day) and methotrexate (25 mg/week), presented to us with worsening vision in both eyes. Her best corrected visual acuity (BCVA) was perception of light in her right eye and counting fingers close to face in her left eye. A slit lamp examination showed an anterior chamber (AC) reaction (1+) in both eyes with posterior synechia, a total cataract in her right eye, and pseudophakia in her left eye. The left fundus showed vitritis, vitreous membranes, chorioretinitis, multifocal areas of retinitis, and retinal vascular sheathing. A systemic examination showed extensive multifocal areas of tinea corporis on the hands and torso. Owing to the leukocytosis (22,000 cells/mm^3^), diagnostic vitrectomy was initially deferred and 100 mg of oral itraconazole was given twice a day for 3 months. The vitritis improved a little and her total white blood cell (WBC) count improved with treatment of the skin infection. Following a diagnostic vitrectomy later in her left eye, resolving areas of retinitis were seen. Complete resolution of eye inflammation was seen at the end of 6 weeks. At the 6-month follow-up, her BCVA was 6/18 in left eye and she was off oral steroids and methotrexate, with no recurrence of inflammation. We speculate a probable association between the ocular inflammation and extensive tinea corporis based on the therapeutic response to itraconazole.

## Introduction

Tinea corporis is a superficial fungal skin infection of the body caused by dermatophytes. Dermatophytes are superficial fungi that infect and replicate within keratinized tissues (1). Ocular involvement is usually limited to the eyelids (2). In this case report we report a rare case of retinal vasculitis with retinitis and panuveitis secondary to an extensive tinea corporis.

## Case description

A 40-year-old women presented to us with worsening of vision in both eyes. She was diagnosed as having idiopathic panuveitis 3 years ago and had been on oral steroids (20 mg/day) and methotrexate (25 mg/week) since then. Her records stated that she had never been able to reduce the oral steroids below 20 mg/day because of recurrences of inflammation and that she had also received multiple intravitreal triamcinolone acetonide injections in both eyes. She had undergone cataract surgery in the left eye 2 years ago with no improvement in vision.

**Figure 1:**
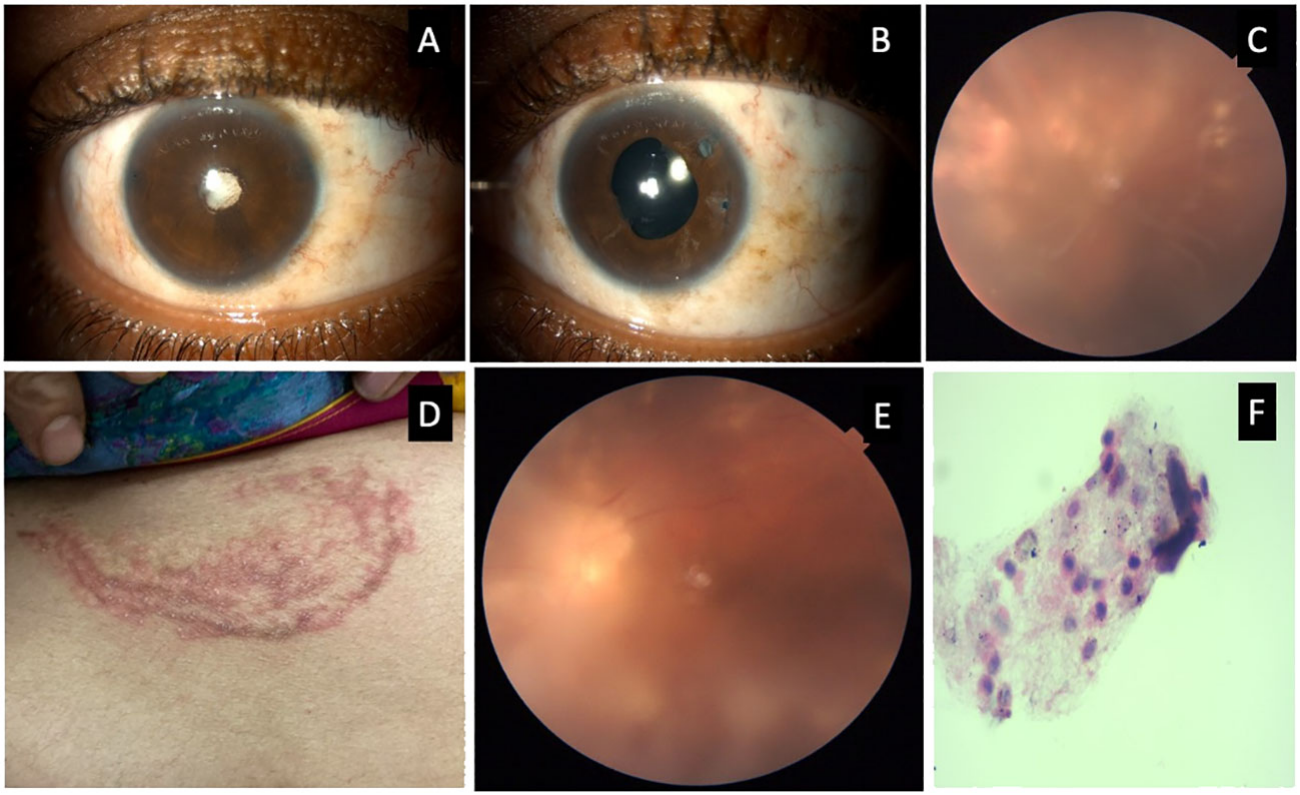
Composite photograph showing slit lamp photograph with a total cataract in the right eye **(A)** and pseudophakia in the left eye **(B)**; a color fundus photograph of the left eye showing vitritis, vitreous membranes, and multiple areas of retinitis with sheathing of arteries and veins **(C)**; external photograph showing annual erythematous lesions on the abdomen **(D)**; a fundus photograph of left eye 1 week after the initiation of systemic antifungals showing decreased vitreous haze **(E)**; and cytology of the vitreous biopsy showing a few lymphocytes and macrophages **(F)**.

**Figure 2:**
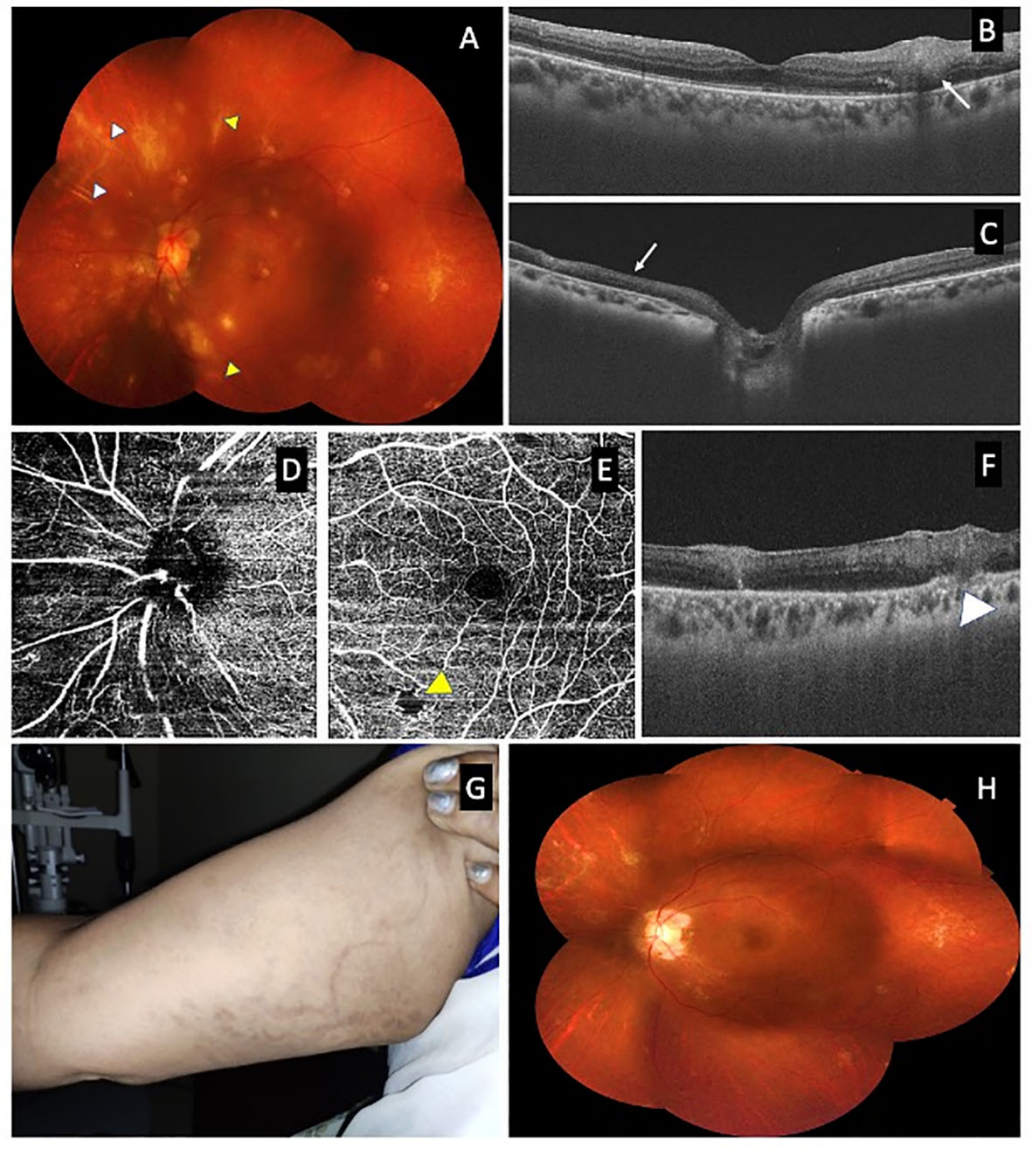
Composite photograph showing a fundus photograph of left eye 1 month after vitrectomy **(A)** with resolving retinitis with sheathing around the arteries (white arrow head) and veins (red arrow head); optical coherence tomography (OCT) over the retinitis **(B)** inferior to the fovea showing hyperreflectivity of the retina (white arrow); OCT over the healed retinitis lesion nasal to the disc showing disorganization of the inner retinal layer **(C)**; OCT angiogram showing a flow void area nasal to the disc corresponding to area of healed retinitis **(D)** and at the macula showing a flow void area (yellow arrow head) corresponding to area of retinitis **(E)**; OCT over the healed retinitis lesion superior to macula showing hyperreflectivity of the retina and involvement of choroid **(F)**; an external photograph showing healed skin lesions over the hand with hyperpigmentation at the 6-month follow-up **(G)**; and a fundus photograph of left eye at the 6-month follow-up showing resolved inflammation and chorioretinal scars.

On examination, her best corrected visual acuity (BCVA) was perception of light in her right eye and counting fingers close to the face in her left eye. A slit lamp examination showed an anterior chamber reaction (1+, standardization of uveitis nomenclature classification) in both eyes. The right eye showed posterior synechia and a total cataract, while the left eye was pseudophakic. The right eye fundus could not be visualized, while the left eye fundus examination showed a grade 3+ vitreous haze (National Institutes of Health grading system) with 4+ vitreous cells (Multicenter Uveitis Steroid Treatment Trial grading system), multiple retinitis patches, and sheathing of both arteries and veins (predominantly arterial involvement). Her intraocular pressures in each eye was in the normal range. Ultrasonography (B scan) of the right eye showed a few echoes in the vitreous cavity, attached retina, and normal choroidal thickness. A systemic examination showed extensive circular and oval erythematous lesions with central clearing and centrifugal spread, raised edges, and peripheral scaly lesions involving the dorsal aspect of her arms, hands, and torso. The patient had had skin lesions for the previous 3 years. However, it was not extensive and the lesions were on her thorax and abdominal regions, which she had not confided to the treating ophthalmologists. However, the skin lesions had worsened significantly with a lot of pruritus 1 year ago. She was diagnosed as having an extensive tinea corporis by the dermatologist. Laboratory investigations showed an increase in total white blood cells (WBCs) (22,000 cells/mm^3^, range 4,000–10,000 cells/mm^3^). Her serum angiotensin-converting enzyme (ACE) levels, blood sugar level, liver function, and renal function tests were normal. Her Mantoux test, venereal disease research laboratory (VDRL) test, *Treponema pallidum* hemagglutination assay (TPHA), and QuantiFERON-TB Gold blood test were all negative. Diagnostic vitrectomy was deferred initially owing to the leukocytosis and extensive skin infection. She was started on 100 mg of oral itraconazole twice daily with a topical ketoconazole (2%) skin cream. Oral steroids were tapered and stopped over 4 weeks. Her skin lesions had started healing at 1 week with a marginal improvement in vision [BCVA 2/60 in the left eye] and a decrease in vitreous haze in left eye to 2+ with better appreciation of retinal details. (**Figure 1**) The total WBC counts also decreased to 12,000 cells/mm^3^. A diagnostic vitrectomy in left eye was done after 2 weeks. Cytology showed few lymphocytes and macrophages, which was suggestive of chronic inflammation. Microbiology [Gram staining, potassium hydroxide (KOH) stain, cultures, and PCR analysis] was negative for bacteria, fungi, and viruses. After 4 weeks, her BCVA had improved to 6/24 in left eye, with healing retinochoroiditis lesions and peripapillary atrophy. (**Figure 2**) The antifungal medications were continued for 3 months, and the dose of methotrexate was reduced to 15 mg/week. At 6 months, her BCVA in left eye was 6/18, the skin lesions had resolved completely, with minimal hyperpigmentation, and the fundus in left eye had healed atrophic areas of multifocal chorioretinitis. Optical coherence tomography (OCT) over the healed retinitis lesions nasal to the disc showed disorganization of the retinal inner layer. OCT angiography showed flow void areas nasal to the disc corresponding to the area of the healed retinitis. The methotrexate was eventually discontinued over 6 months. At 2 years’ follow-up, she is doing well with no recurrences of ocular inflammation.

## Discussion

Various organisms, such as *Toxoplasma*, cytomegalovirus, herpes simplex virus, rubella virus, *Mycobacterium tuberculosis, Toxocara, Treponema pallidum, Bartonella*, Candida spp., and *Coccidioides*, are known to cause infective retinochoroiditis (3–6). Fungi, such as *Trichosporon* and *Sporotrichum schenkii*, involving the retina and choroid have been reported (7, 8).

Tinea corporis is a common fungal infection seen in the developing world. Though our case was initially diagnosed as idiopathic panuveitis, the presence of extensive skin involvement as a result of this infection with elevated leukocyte counts and the refractory nature of panuveitis led us to speculate on an association between the ocular issues and the skin infection. The effective therapeutic response to antifungals and the resolution of the ocular inflammation strengthened our speculation. Agarwal et al. (9) reported a case of unilateral chorioretinitis secondary to a tinea corporis, and Kawali et al. (10) reported two cases of choroiditis with occlusive vasculitis secondary to tinea corporis. Contrary to these reports, our case had vitritis, vitreous membranes, multifocal areas of chorioretinitis, retinitis, and retinal vasculitis with involvement of both arteries and veins. Though neither the PCR analysis, nor the culture of the vitreous showed any positivity for fungal infection, the excellent therapeutic response achieved with systemic antifungals and the discontinuation of methotrexate confirms the association between the skin and ocular lesions.

This case report highlights an interesting ocular involvement in extensive tinea corporis and adds to the existing literature on the presence of ocular involvement in this type of skin infection.

## Patient perspective

At the time of presentation, the patient was visually disabled because of bilateral panuveitis and no improvement in vision despite undergoing treatment for the past 3 years. On further examination, a diagnosis of an extensive tinea corporis skin infection was made. Initiation of systemic antifungal therapy along with vitrectomy improved her skin infection and ocular inflammation, thereby improving her quality of life.

## Data availability statement

The original contributions presented in the study are included in the article/supplementary material. Further inquiries can be directed to the corresponding author.

## Ethics statement

Written informed consent was obtained from the individual(s) for the publication of any potentially identifiable images or data included in this article.

## Author contributions

VK and KB were involved in the concept and design of study, acquisition of data and analysis, interpretation of data, and drafting the article. KB revised the article critically for important intellectual content. The manuscript has been read and approved by all the authors and the requirements for authorship have been met, and each author believes that the manuscript represents honest work and the information is not provided in another form. All authors contributed to the article and approved the submitted version.
